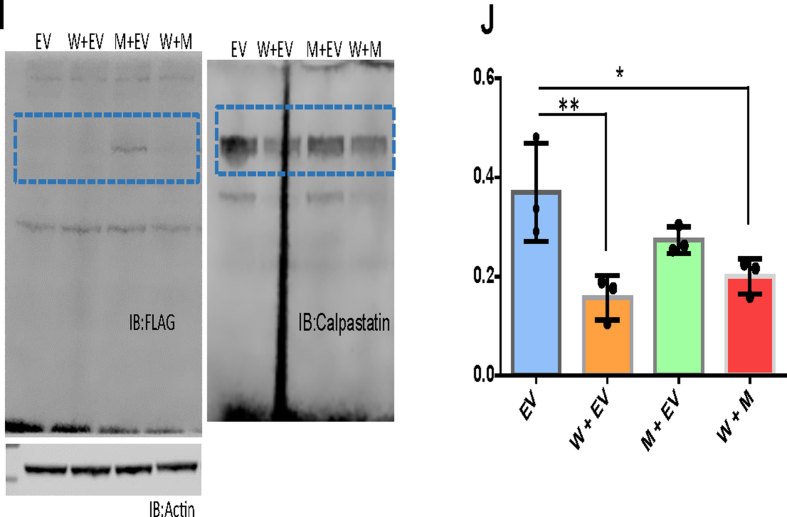# The 21-base pair deletion mutant Calpain3 does not inhibit wild-type Calpain3 activity

**DOI:** 10.1016/j.gendis.2024.101301

**Published:** 2024-04-08

**Authors:** Swati Maitra, Seungjae Oh, Yun-Jeong Choe, JiHye Kim, Nam Chul Kim

**Affiliations:** Department of Pharmacy Practice and Pharmaceutical Sciences, College of Pharmacy, University of Minnesota, Duluth, MN 55812, USA

Calpain3 is one of the calpain protease family members that are predominantly expressed in skeletal muscle. Loss-of-function mutations in Calpain3 have been related to autosomal recessive limb-girdle muscular dystrophy 1 (LGMDR1), a common form of muscular dystrophy. Recently, the heterozygous 21-bp deletion mutation of the Calpain3 gene has been reported to cause autosomal dominant limb-girdle muscular dystrophy 4 (LGMDD4) which suggests that the mutant proteins act in a dominant-negative manner. Therefore, we examined whether the mutant protein could suppress the activity of wild-type Calpain3 using a cell culture model and a *Drosophila* model. The mutant Calpain3 resulted in catalytic inactivation, which did not inhibit wild-type Calpain3 autolytic and catalytic activities in HeLa cells. Overexpression of wild-type and mutant Calpain3 in *Drosophila's* eyes and muscles did not exhibit significant dominant toxicity. We provide evidence that mutant Calpain3 does not suppress wild-type Calpain3 activity. Rather, it is a mutant lacking autocatalytic activity like many other loss-of-function Calpain3 mutants causing LGMDR1. Our results implicate that the stability of the heteromeric mutant and wild-type Calpain3 complexes may be affected without inhibiting the wild-type activity *per se*. However, a thorough investigation is necessary to understand the molecular mechanism and dominant inheritance of the heterozygous 21-bp deletion mutation in LGMDD4.

Limb-girdle muscular dystrophy (LGMD) is a group of neuromuscular diseases characterized by progressive muscle weakness and muscular atrophy of the proximal muscles caused by a defect in Calpain3.^1^ Calpain3 is a member of the Calpain protease family, which are non-lysosomal calcium-regulated proteolytic enzymes predominantly found in the skeletal muscles.^1^ Calpain3 forms an integral part of the sarcoplasmic reticulum of the skeletal muscles and plays a role in maintaining muscle integrity and function by regulating sarcomeric protein turnover and maintaining the integrity of the sarcomere structure.^2^ LGMDR1 (previously referred to limb-girdle muscular dystrophy type 2A) is caused by a recessive mutation in the Calpain3 (*CAPN3*) gene.^1^ However, Vissing and colleagues reported 37 patients from 10 European families with an autosomal dominant LGMD co-segregating with an in-frame 21-bp deletion (c.643_663del21, p. Ser215_Gly221del) in *CAPN3*.^3^ Nine affected patients had a milder phenotype than those affected by LGMDR1, with normal expression of the mutated mRNA and no evidence of nonsense-mediated mRNA decay, but there was a significant decrease (<15% of control values) at protein levels measured by Western blot analysis. Therefore, the authors proposed a dominant-negative effect of the *CAPN3* deletion by inhibiting wild-type Calpain3 activity by mutant Calpain3 when they formed homomeric complexes.^3^ Another group also reported the autosomal dominant inheritance of LGMD with the *CAPN3* 21-bp deletion independently.^4^ In this study, we sought to understand the phenomenon of the dominant toxicity of the 21-bp deletion mutation of Calpain3 by examining catalytic activity and dominant phenotypic aberration using a human cell line and *Drosophila*, respectively.

To test the expression and stability of wild-type and deletion mutant Calpain3, we transiently transfected C-terminal FLAG-tagged wild-type and 21-bp deletion mutant Calpain3 plasmid constructs to HeLa cells followed by immunoblotting (Fig. 1B, detailed methods in supplementary data). Unexpectedly, the overexpression of FLAG-tagged wild-type was not detected at the expected molecular weight of its full-length protein in the immunoblot by anti-FLAG antibody, but there was a prominent expression of full-length band of FLAG-tagged deletion mutant protein (Fig. 1B, left panel). Consistently, the anti-Calpain3 antibody also detected an intense overexpressed band in the mutant lane but not in the wild-type and empty vector lanes (Fig. 1B, right panel). Calpain3 exhibits extremely rapid and exhaustive autolysis.^2^ Thus, this result may indicate that the wild-type undergoes its unique autolysis but the mutant lacks the autolytic activity. This also implies that a faint band close to the molecular weight of Calpain3 (94 kDa) was detected in the empty vector and wild-type lanes which may be a stabilized form of Calpain3 with unknown mechanisms in HeLa cells. Typically, two fragments are generated upon their autolysis around 30 kDa and 60 kDa. Since we used Calpain3 antibody detecting a peptide sequence locating the N-terminal part and transfected Calpain3 has a C-terminal FLAG-tag (Fig. 1A), a smaller N-terminal (∼30 kDa) and a larger C-terminal autolyzed fragments (∼60 kDa) can be detected by anti-Calpain3 and anti-FLAG antibodies, respectively. Therefore, we examined whether two fragments were detected. Although we detected a 30 kDa band with an anti-Calpain3 antibody, the 60 kDa band was not clear due to non-specific backgrounds (Fig. S1A). However, after modifying a few parameters (detailed methods in supplementary data), we clearly detected both bands in wild-type but not in mutant (Fig. 1C). Therefore, transiently transfected Calpain3 (wild-type) was rapidly auto-degraded and their full-length protein could not be detected by anti-FLAG and anti-Calpain3 antibodies in repeated experiments (Fig. 1B, C; Fig. S1B) but the intermediate autolyzed fragments were successfully detected (Fig. 1C, D; Fig. S1B–D and S2A–F). Thus, the 21-bp deletion mutant protein is an inactive form of Calpain3 for its autolysis.

**Figure 1:**
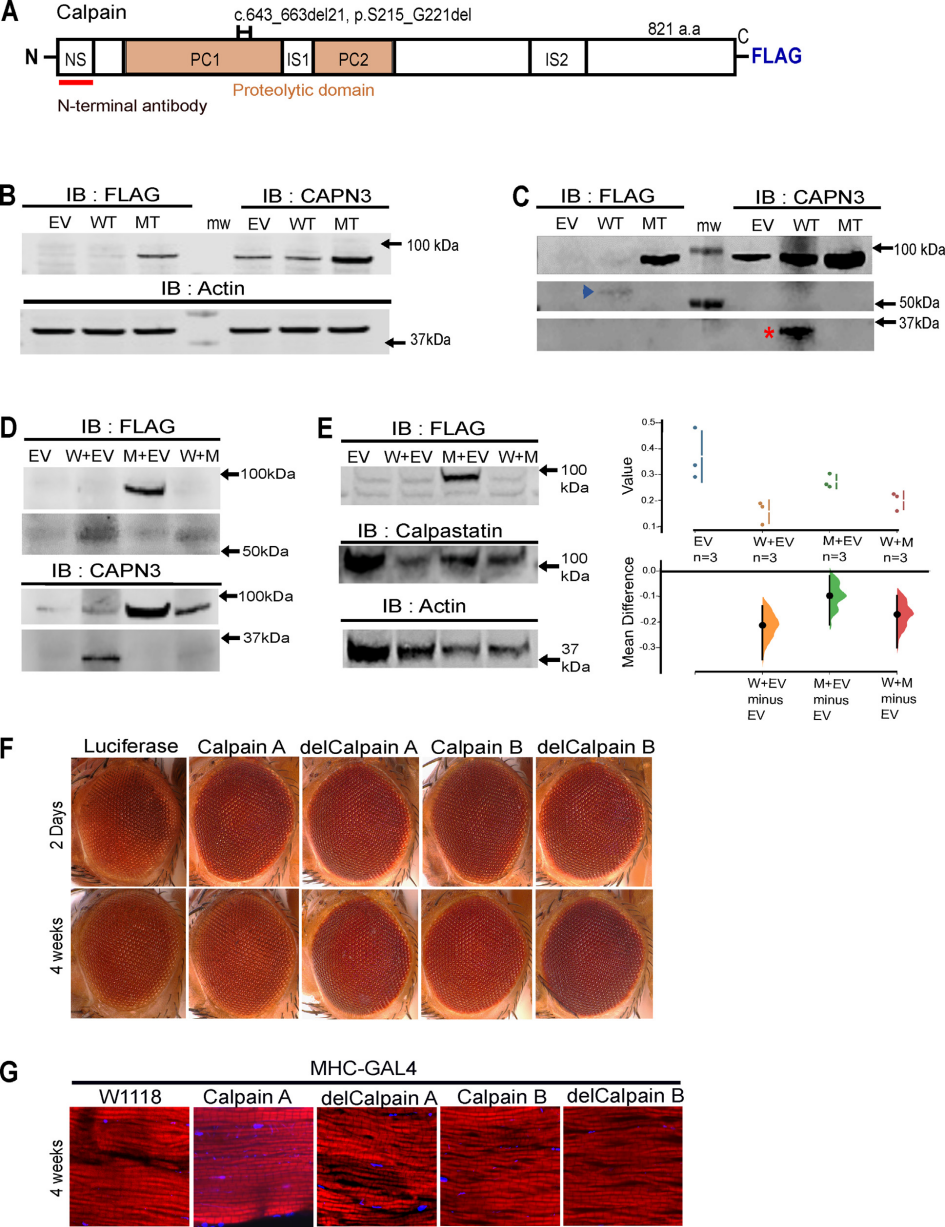
The 21 bp deletion mutant (MT) Calpain3 does not inhibit wild-type (WT) Calpain3 activity. The deletion MT Calpain3 loses its catalytic activity: **(A)** Schematic diagram of *C*-terminal FLAG-tagged Calpain3 and deletion mutation, a 21-bp (7 amino acids) deleted region, with *N*-terminal anti-Calpain3 antibody binding site (marked in a black bracket and a red line), respectively. NS, N-terminal leader sequence; PC1 & 2, protease core1 & 2; IS1 & 2; insertion sequence1 & 2. The deletion MT Calpain3 does not inhibit the activity of WT Calpain3: **(B)** Immunoblotting with anti-FLAG, anti-Calpain3, and anti-Actin (loading control) antibodies on overexpression of empty vector (EV), WT, and MT Calpain3 in HeLa cells. FLAG-tagged MT was detected but not WT (Left panel). An intense MT band and faint bands in WT and EV lanes were detected when blotted with an anti-Calpain antibody (Right panel). **(C)** Immunoblotting for detection of autolyzed fragments of Calpain3. FLAG-tagged WT Calpain3 was rapidly processed by its own autocatalytic activity (the blue arrowhead indicates the C-terminal larger fragment and the red asterisk indicates the smaller N-terminal fragment) but none in the case of the MT lane. **(D)** Immunoblotting with anti-FLAG and anti-Calpain3 antibodies upon co-expression of WT and MT. WT Calpain3 was normally autolyzed in both WT and EV lanes in (W + EV) and also along with the deletion MT co-expression lane (W + M). The MT proteins were also processed and detected by an anti-Calpain3 antibody. **(E)** Immunoblot for calpastatin expression detected by anti-calpastatin antibody. Calpastatin was also processed with WT/MT co-expression at a comparable level to WT overexpression (left panel). Quantification with three independent replicates for two-sided permutation *P* values: 0.0, 0.127, and 0.0, respectively (right panel). The deletion MT of *Drosophila* CalpainA and CalpainB do not have dominant toxicity: **(F)** WT and deletion MT CalpainA and CalpainB were expressed in *Drosophila's* eyes with glass multimer reporter-GAL4. In two days (upper panel) and four-week-old flies (lower panel), no significant developmental and age-related dominant toxicity was observed (females, at 25 °C). **(G)** Representative immunohistochemical images of the thoracic segment of four-week-old flies showed no significant muscle degeneration and related abnormalities when WT and MT CalpainA and CalpainB were expressed in *Drosophila's* muscles with myosin heavy chain-GAL4. (red: phalloidin, myofibrils; blue: DAPI, nucleus).

Vissing et al proposed that mutant Calpain3 may dominant-negatively interfere with the activity of wild-type Calpain3. We tested this hypothesis by co-transfecting with both wild-type and deletion mutant constructs (Fig. 1D; Fig. S1C, D, S2C–F). We examined whether mutant Calpain3 inhibited the autolytic activity of the wild-type Calpain3 and also its catalytic activity against calpastatin which is a well-known substrate for Calpain3. As seen in Figure 1D, autolysis of wild-type Calpain3 was observed in the wild-type and empty vector lanes. Corresponding fragments were also detected with anti-FLAG and anti-Calpain3 antibodies as expected. Interestingly, when the mutant protein was co-expressed with wild-type protein, the expression of Calpain3 decreased significantly compared with when the mutant protein was expressed alone (Fig. 1D; Fig. S1C, D, S2C–F). This indicates that inactive mutant Calpain3 was processed by wild-type Calpain3, and the intramolecular and intermolecular autocatalytic activity of wild-type Calpain3 was not reduced by the Calpain3 mutant. When calpastatin levels were used as a readout of wild-type Calpain3 activity, calpastatin was decreased in the wild-type and empty vector lanes compared with the empty vector-transfected control lane whereas there was no change in the calpastatin level in the mutant and empty vector lanes (Fig. 1E). When wild-type and mutant were co-expressed, the calpastatin level was similarly decreased to that observed in the wild-type and empty vector lanes, suggesting that the mutant form could not block the wild-type catalytic activity against its substrate significantly. This phenomenon was observed in three independent replicates and was statistically significant (Fig. 1E; Fig. S1E, F, S2G–J). These results, altogether, demonstrated that the deletion mutant did not inhibit wild-type Calpain3 autolysis and catalytic activity against another substrate.

For *in vivo* studies, we generated a *Drosophila* version of the 21-bp deletion on the two *Drosophila* homologs of human Calpain3, CalpainA, and CalpainB, and their deletion mutant counterparts, delCalpainA and delCalpainB (Fig. S1G). To test whether the overexpression of these constructs could cause dominant toxicity, we first expressed CalpainA, delCalpainA, CalpainB, and delCalpainB in *Drosophila's* eyes, which are not expected to express endogenous CalpainA and CalpainB, with the glass multimer reporter-GAL4 driver. None of them caused significant abnormal eye phenotypes at eclosion and at four weeks old (Fig. 1F). To better investigate the function of mutant Calpain3 that may act in a dominant-negative manner, we expressed CalpainA, delCalpainA, CalpainB, and delCalpainB in *Drosophila's* muscles using the myosin heavy chain-GAL4 driver. Immunohistochemical analysis of thoracic indirect flight muscle sections of flies overexpressing wild-type and mutant CalpainA and CalpainB did not show significant muscle degeneration or sarcomere structure abnormality in aged flies (4 weeks old) (Fig. 1G). These findings indicate that the dominant toxicity of 21-bp deletion cannot be recapitulated in either *Drosophila's* eyes or muscles.

We have provided here experimental evidence to show that the deletion mutation of Calpain3 causes loss of autolysis activity and does not inhibit the catalytic activity of its wild-type counterpart, and can be normally processed by the wild-type Calpain3. Our results strongly support that the impaired stability of Calpain3 homomeric complexes may be the key to understanding the decreased Calpain3 levels (<15%) in patients. Calpain3 protein was reported to form a trimeric protein recently.^5^ If only wild-type homotrimeric complexes are stable and others containing a mutant subunit are not, then only 12.5% of Calpain3 can be stably maintained in patients. This corresponds well with the levels of Calpain3 in patients. A detailed biochemical analysis is required to understand how mutant Calpain3 affects the stability of complexes without affecting the wild-type activity itself. In addition, it may be necessary to examine the impact of the deletion mutant Calpain3 at other developmental stages of muscle differentiation in *Drosophila* and/or study the effect of the mutant Calpain3 using human myoblasts or differentiated myotubes to gain more insight.

## Author contributions

N.C.K. conceived the project. Y-J.C., S.J.O., S.M., and J.H.K. designed and performed the experiments. Y-J.C., S.J.O., S.M. analyzed the data. S.M., S.J.O., and N.C.K. wrote the manuscript.

## Conflict of interests

The authors have no conflict of interests to disclose.

## Funding

This research was supported by grants from the 10.13039/100005202Muscular Dystrophy Association and the Wallin Neuroscience Discovery Fund and the Engebretson Drug Design and Discovery Fund (to Nam Chul Kim).

## Figures and Tables

**Figure S1:**
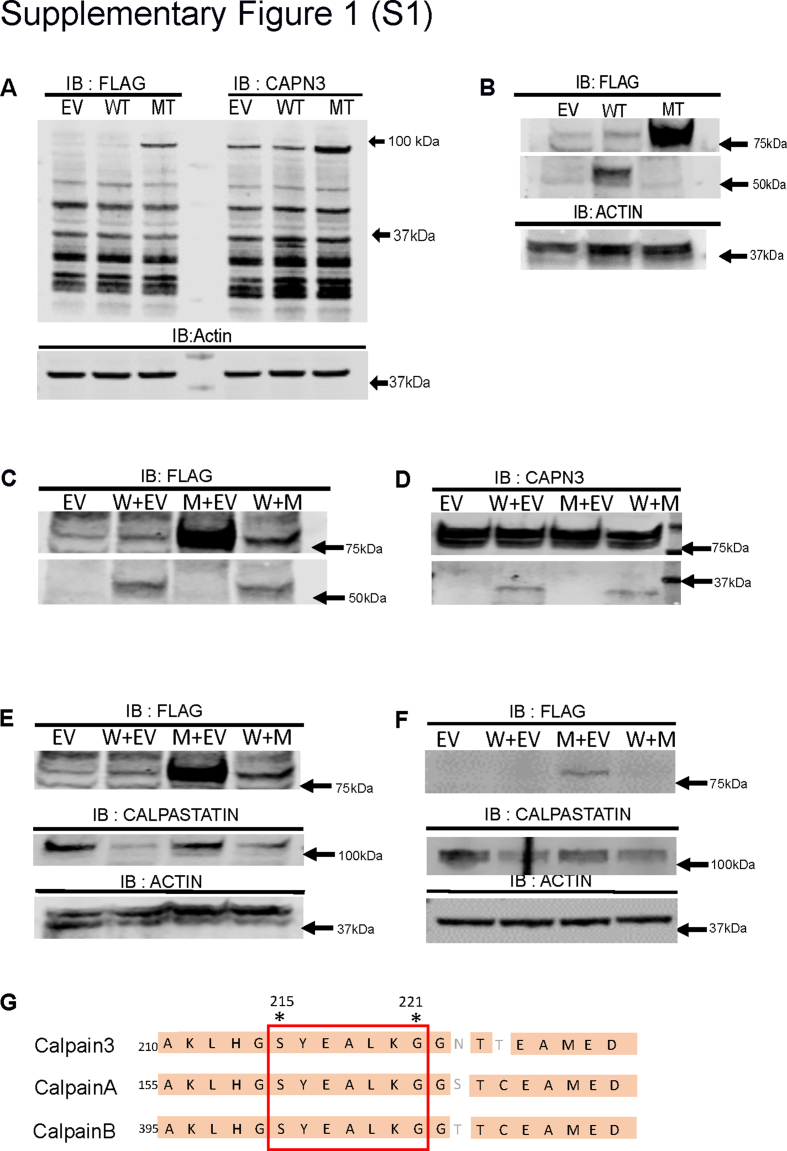


**Figure S2:**
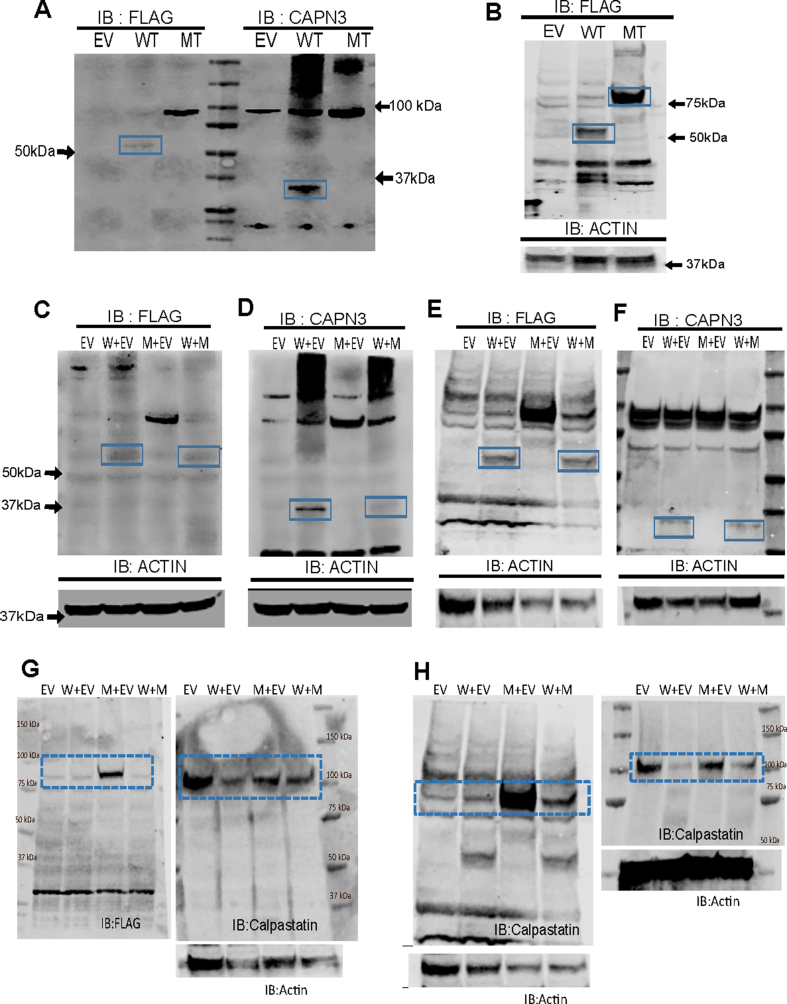


**Figure S3:**