# Supplementary material for: The 21-base pair deletion mutant Calpain3 does not inhibit wild-type Calpain3 activity
Source: Genes Dis. 2024 Apr 8;12(1):101301. doi: 10.1016/j.gendis.2024.101301 (PMC11472601; doi:10.1016/j.gendis.2024.101301)
Supplement: Multimedia component 1 [file mmc1.docx]

**Supplementary Figure 1 (S1) The protein expression levels of Wild-type and deletion mutant Calpain3.**

1. Whole immunoblot for detection of autolyzed fragments of Calpain3. Actin was kept as a loading control. **(B)** Immunoblot showing FLAG-tagged C-terminal fragment (~60 kDa) of Wild-type Calpain3 was clearly visible when probed with anti-FLAG antibody but none in the case of mutant lane (MT). **(C)** **(D)**Immunoblots showing the detection of N-terminal **(C)** and C-terminal **(D)** autolyzed fragments of Calpain3 in Empty vector (EV), Calpain3 wild-type (WT), and Calpain3 deletion mutant (MT) transfected HeLa cell lysates. Please note that this **(C)** was an independent experiment from **(D)** and the blot **(D)** was overexposed in order to have a clear view of the hard-to-detect smaller fragments in W+EV and W+M lanes. **(E) (F)** Immunoblotting for detection of Calpastatin expression in Empty vector (EV), Calpain3 wild-type (W+EV), and Calpain3 deletion mutant (M+EV) and (W+M) co-transfected HeLa cell lysates. Actin was kept as a loading control (Quantification and statistical results are in Figure 1E and Supplementary Information SI). **(G)** Amino acid sequence alignment around 21-bp deletion region across Human Calpain3 and *Drosophila* CalpainA and B. The conserved sequences are shown relative to the positions of the translation start codon (indicated by bold letters with colored backgrounds). All the seven amino acids in the deleted regions are highly conserved across the species (marked with a red box).

**Supplementary Figure 2 (S2) Whole immunoblots of wild-type and deletion mutant Calpain3 overexpression in Hela cell**.

(A) Immunoblots showing detection of autolyzed fragments- C-terminal (~60 kDa) and N-terminal (~30 kDa) fragments for Figure 1C. The bands were highlighted in blue box for all the blots. (B) Whole Immunoblot for Figure S1 B. (C) (D) (E) (F) Whole immunoblots for detection of full-length and autolyzed fragments in co-expression experiments for Figures 1D and S1 C, D. (G) (H) (I) Whole immunoblots for detection of Calpastatin expression levels for Figures 1E and S1 E, F. (J) Graphical representation of additional statistical analysis (ANOVA - Dunnett’s test) for Figures 1E and S1E, F.

**METHODS**

**DNA constructs**

The human Calpain3 and 21 bp-deletion Calpain3 mutant were synthesized and sub-cloned in the pcDNA3.1 vector containing a C-terminal FLAG tag by GenScript. Wild-type and deletion mutant plasmid constructs for *Drosophila* CalpainA and CalpainB were synthesized and sub-cloned in the pUASTattb vector to generate transgenic *Drosophila* lines from BestGene.

**Cell culture and transfection**

HeLa cell lines were purchased from the American Type Culture Collection (ATCC). Cells were maintained in Dulbecco-modified Eagle medium (Gibco) supplemented with 10% fetal bovine serum (Gibco), 1× penicillin/streptomycin (Invitrogen), and 1 x GlutaMax (Gibco). Cells were transfected using jetPRIME (Polyplus) transfection reagent according to the manufacturer’s protocol. For plasmid transfections, around 1X10^6^ cells on were plated on a 60mm tissue-culture dish and allowed to grow for 24 hrs. Transfections were carried out with 5 ug of plasmid and 10ul of jetPRIME transfection reagent (1:2) mixed in 250uL of jetPRIME buffer per dish. In cases of co-transfections, plasmids were mixed at equimolar concentrations. After 24 hrs of the transfection, cells were harvested and lysed in ice-cold RIPA Buffer.

**Immunoblotting** **and Antibodies**

Cell samples were collected and lysed in ice-cold RIPA buffer (Cell Signaling Technology) with protein inhibitor cocktail (Sigma) and subsequently loaded in equal amounts (30 ug of protein /lane) and separated by sodium dodecyl sulfate-polyacrylamide gel electrophoresis (SDS-PAGE) after measuring protein concentration by bicinchoninic acid (BCA) (Pierce). The immunoblots were incubated in Blocking buffer (LI-COR Biosciences) for 1 hr at room temperature followed by antibody incubation. The following primary antibodies were used: FLAG (Sigma and Proteintech, 1:1000), Calpain3 (Proteintech and Santa Cruz Biotechnology, 1:1000), Calpastatin (Proteintech and Santa Cruz Biotechnology, 1:1000), Actin (Proteintech and Santa Cruz Biotechnology, 1:5000). Immunoblots were visualized and analyzed with the Odyssey FC System (LI-COR).

**Generation of Drosophila lines**

Transgenic *Drosophila* lines were generated by BestGene with the standard PhiC31 integrase-mediated transgenesis system. All transgenes were inserted into the third chromosome attp2 site to avoid positional gene expression differences. Fly cultures and crosses were performed on standard fly food (Genesee Scientific) and raised at 25°C with a 12:12 hr light: dark cycle. GMR-GAL 4 and MHC-GAL4 were used as drivers for their expression in the eye and muscles, respectively.

**Drosophila adult muscle preparation and immunohistochemistry**

To assess the muscle integrity and sarcomere structure of the indirect flight muscle, adult flies were quickly dissected and fixed with 4% paraformaldehyde in 1X PBS for 1 hour, embedded in OCT compound (Fisher Scientific), and snap frozen in liquid nitrogen or dry ice. 15-16 microns thick sections were cut by a cryomicrotome (Leica) and directly mounted on the slide. Additional fixing was performed with 4% paraformaldehyde in 1XPBS for 10 minutes at room temperature prior to permeabilization with 0.2% Triton X-100 buffer in PBS and blocked with 5% BSA solution in PBS for 1 hour. Sections were incubated with phalloidin–Alexa Fluor 594 (Invitrogen) overnight at 4°C for muscle staining and finally were mounted with Prolong Diamond Antifade Reagent with DAPI and imaged with an LSM 710 confocal microscope (Carl Zeiss) with 63× magnification.

**Statistical Analysis**

Graphs for the quantitative analysis of immunoblots (mean difference between groups with 95% confidence interval) were generated using Estimation Stats, <http://estimationstats.com> (Bernard, 2021; Ho et al., 2019). We performed an unpaired T-test for the evaluation of our data with a two-sided permutation test to calculate p-value. P-value (probability) is a test for significance of a particular group of data. Additionally, ANOVA with Dunnett’s multiple comparison test was also performed and was found to be statistically significant among the same groups. The details for statistical evaluation and the raw data are included in the Supplementary Information (SI).

**References**

1. Bernard, C. (2021). Estimation statistics, one year later. In *eNeuro* (Vol. 8, Issue 2). Society for Neuroscience. <https://doi.org/10.1523/ENEURO.0091-21.2021>
2. Ho, J., Tumkaya, T., Aryal, S., Choi, H., & Claridge-Chang, A. (2019). Moving beyond P values: data analysis with estimation graphics. In *Nature Methods* (Vol. 16, Issue 7, pp. 565–566). Nature Publishing Group. https://doi.org/10.1038/s41592-019-0470-3
